# Supranational Assessment of the Quality of Probiotics: Collaborative Initiative between Independent Accredited Testing Laboratories

**DOI:** 10.3390/microorganisms9071456

**Published:** 2021-07-07

**Authors:** Jean-Pol Warzée, Marina Elli, Abdoulaye Fall, Daniela Cattivelli, Jean-Yves François

**Affiliations:** 1 ESLP–European Scientific League for Probiotics, 1472 Genappe, Belgium; 2 AAT-Advanced Analytical Technologies Srl, Fiorenzuola d’Arda, 29017 Piacenza, Italy; marina.elli@aat-taa.eu (M.E.); daniela.cattivelli@aat-taa.eu (D.C.); 3 Genalyse Partner, a Food Chain ID Company, 4040 Herstal, Belgium; afa@genalyse.be; 4 Quality Partner, a Food Chain ID Company, 4040 Herstal, Belgium; jyf@quality-partner.be

**Keywords:** quality, consumer, end-user, probiotics, food supplement, metagenomic, label, qPCR, accreditation

## Abstract

Recent acquisitions about the role of the microbiota in the functioning of the human body make it possible to envisage an increasing use of beneficial microbes, and more particularly of probiotics as well as their metabolites, as nutritional supplements. National and EU authorities are engaged in assuring the safety and quality of food supplements and in defining rules to assess and communicate their efficacy on human health. The quality of probiotics, intended as strains’ identification, viability, and stability over time, is a crucial factor of credibility with consumers and health professionals. Analytical technologies for the quality control of probiotics must also be adapted to new preparations, such as those including new multistrains complex combinations. Accredited laboratories face this relevant challenge on a daily basis. Through its close collaboration with the laboratory commissioned to produce the specifications for its ESLP quality label (identification and quantitative analyses) together with its scientific committee, the ESLP has been focusing on this issue for 10 years. Recently, as part of the internationalization of the ESLP quality label, a new and unique initiative in Europe for the evaluation of the quality of probiotic preparations has been carried out. The collaboration between two accredited laboratories in Belgium and in Italy represented a concrete example of supranational collaboration in the assessment of the quality of probiotic preparations. Results show that both laboratories are in line as expected in terms of performance. Common approaches to the qualitative assessment of probiotic preparations, especially for complex and composite recipes, in terms of number of strains and included substances, should be encouraged and promoted all over the EU.

## 1. Quality of Probiotic Preparations

Derived from a Greek word, the word “probiotic” is meaning “for life” [1], and its definition is based on an expert group consensus statement: “live microorganisms that, when administered in adequate amounts, confer a health benefit on the host” (FAO/WHO Expert Group 2001) [2]. Although the mechanisms by which they confer these benefits remain largely unclear [3], the probiotic category is generating from the consumers in Europe and outside Europe a growing interest in the different areas of health: digestive, immunological, respiratory health. For probiotics, being live microorganisms and most of their benefits being highly strain-specific [1,3,4], product quality is key.

Quality is perceived by consumers as a multifactorial attribute. Many factors, such as compliance, functions, process, and composition, contribute together to affect the final perception of a commercial product. Composition, intended as identification, purity, viability and stability, is surely one of the leading aspects of the quality of probiotic preparations. Several indicators are available to manage and therefore communicate quality. Product certification and labeling, adoption of quality standards, and/or quality systems are usually considered among valid quality indicators.

Based on IPA Manifesto (International Probiotics Association Europe; www.ipaeurope.org, access date 15 January 2021) [5], the European probiotic supplement market in 2019 was close to a third of the global consumption, with Italy being the largest market for probiotic supplements in Europe and the second largest market size in the world, second only to the US.

In Europe as well as around the world, quality of probiotic preparations is arising increased attention due to the requirement for a qualification of probiotics with doctors and clinicians able to drive healthy messages to consumers and patients. In a very composite market, such that of probiotic food supplements, it is becoming more and more relevant to qualify the originality of a product and to find key information to impress the audience. The present era in which “the more the better” concept is gaining increasing success, through the idea that multi-strain high-dosage probiotics are thought to be more efficient for human health, opens a very challenging time window to promote companies’ awareness on quality issues. A significant lack in transparency and therefore in medical and consumers’ trust in probiotic preparations has been highlighted by several authors in recent years [6,7,8,9,10]. Moreover, surveys on commercial products containing probiotics sometimes contributed to the loss of trust, due to the fact that the identity and number of recovered species did not always correspond to those stated on the labels, despite their significant contributions to the awareness of the stakeholders [11,12,13,14,15].

A generalized call for a revision of the quality concept towards a more comprehensive approach, as well as the availability of tools to valorize beneficial high-quality probiotic preparations, is perceived all around Europe [16,17]

Collaborative initiatives have to be promoted in order to define common approaches to the assessment of the quality of probiotic preparations and therefore overcome, at least from the analytical point of view, the significant fragmentation of the EU regulatory framework. The definition of consensus approaches and shared analytical protocols are essential to build up a common vision to treat the assessment of the quality of probiotic preparations in a reliable manner even for different dosages, for unconventional preparations, and for high-complexity products.

## 2. Regulatory Framework of Probiotic Preparations in Europe

Regulatory standards about probiotics greatly differ among countries. More and more multi-strains probiotics entered the market in the last years.

The strains used in food supplements need to be on the positive QPS list (Qualified Presumption of Safety) granted by the EFSA (European Food Safety Authority). To be granted QPS status, a microorganism must meet the following criteria: taxonomic identity well defined, available body of knowledge sufficient to establish its safety, lack of pathogenic properties established and substantiated, intended use clearly described.

In a recent publication in Frontiers in Medicine, Neunez et al. 2020 underlined the need for the quality and objectivity of information provided online to the public [18].

In the Italian regulatory framework, mainly due to the seniority of the field, the huge number, and the relevant complexity of marketed probiotic preparations, results tend to be more stringent than in other EU countries. Italian Guidelines on Probiotics and Prebiotics, issued by the Ministry of Health starting from 2011 and frequently revised, considers clear parameters stating the quality of finished probiotic products, including safety issues needed to assure the protection of the consumers. The latest revision of March 2018 [19] clearly states the features of probiotics used for human consumption in terms of identification at the species and strain level, minimum number of viable microorganisms to be provided daily to the consumer, and tolerance between the concentration in viable cells declared on the label and that measurable at the end of the shelf life.

Belgium is in the top three countries related to Probiotics Food supplements per capita expenditure. The Belgian authorities are strictly implementing the European legislation related to Food supplements and Probiotics, included the regulation N°1924/2006 on nutrition and health claims made on foods [20]; the commercial use of the word “probiotic” is not allowed neither towards consumers nor towards health-care professionals due to the fact that the “heath benefit” is part of this definition.

Recently, an important decision has been taken by the Spain authorities: in October 2020, the use of the term “probiotic” on the labels of food and food supplements produced and commercialized in the country has been accepted by the Spanish Agency for Food Safety and Nutrition (AESAN) [21] with reference to the mutual recognition principle. So, as from beginning 2021, seven countries—Spain, Italy, Greece, Bulgaria, Poland, the Czech Republic, and Malta—are friendly towards use of the term “probiotics” on food supplement labels, subject to different conditions (www.sandwalkbio.com, access on 10 February 2021); Spain and Italy being two of the three most important probiotic food supplements markets in Europe, both of them representing together 45% of the total probiotics supplements market in Europe (IPA International Probiotics Association 2021).

The recent rapid growth of the e-commerce, registered as from 2019, also participates in changing the rules: data, EUR 108 million of probiotics were bought online by consumers in the EU (excluding UK) in 2019 with nearly 80% including health claims on pack on their website (Lumina Intelligence www.lumina-intelligence.com, access on 10 February 2021).

Indeed, it is now possible also in Denmark to use the word “probiotic” on labels. In March 2021, The Netherlands Food and Drug Administration has released guidance stating that the term “probiotic” is allowed on Food and supplements labels, as mandatory information that characterizes the product.

Besides in order to change the EU regulation to allow use of the word “probiotic” on labels across the board, Denmark has promised by the end of January to work with Spain and other EU countries.

This, together with the recent Spanish decision, will probably create a domino effect with more and more countries joining the group of the here above-mentioned countries.

## 3. Quality Labeling: The ESLP (European Scientific League for Probiotics) Initiative and Expertise

Over the last 10 to 15 years, many probiotic food supplements based on lactobacilli, bifidobacteria, etc., have been introduced in Belgium and Europe. The Probiotics segment today in Europe is leading the Top 20 categories in the Food supplements market and is registering each year robust sales performance.

Founded in 2011, the ESLP—European Scientific League for Probiotics–non-profit association [22], has created the ESLP Quality Label to validate qualified products based on the approach described below. Besides, by way of its Scientific Committee, composed of neutral experts and scientists recognized in the field, the European Scientific League for Probiotics helps to promote scientific research in human health. ESLP is complementary to the other Probiotics International Associations.

The “ESLP label” is a “Quality Label” exclusively granted to specialties sold through the pharmacy channel. The ESLP quality label is assigned at spontaneous application by the manufacturer and is dedicated to documented strains and associations.

The ESLP label focuses on the fact that each strain in the product is specific and includes:

1. Three levels of denomination for a specific strain: genus, species, strain.
2. Registration number in a “strain bank”.

The quality label is assigned after qualitative and quantitative microbiological analyses have been carried out by an independent accredited laboratory: for the Belux, Quality Partner-Genalyse Partner, BELGIUM SA, ex-spin-off of the University of Liège.

The ESLP label certifies that the strains present in the product concerned are specific, viable, and present in sufficient quantities throughout the product shelf-life, in accordance with the information available on the product packaging.

The ESLP label does not provide in any way a quality scale between labeled products nor does it disqualify products that do not wish to participate in the trials proposed for granting the label. It has no health claim value. Any company whose product is labeled undertakes to respect in its communication the limitation rules relating to the strictly specified ESLP-labeled product and a strictly defined geographical area.

The ESLP quality label is exclusively for products distributed in pharmacies, and applications are freely submitted to the ESLP by distributors of lactobacilli, bifidobacteria, etc. strains.

The products are sampled randomly in the market, and the ESLP quality label is granted after qualitative and quantitative microbiological analysis based on the specifications defined by the ESLP Scientific Committee. Analysis criteria of the ESLP label can change based on available criteria issued by authoritative national/international scientific groups.

The purpose is to verify if probiotic bacteria announced by the business operators are present in sufficient quantities in the products sold to consumers.

The product is considered as acceptable according to two criteria:

1. The quantity of bacteria announced is exact: ESLP allows a maximum 1 log difference from the amount stated on the packaging and in the instructions for the proprietary medicinal product for both of these quantitative measures:
  (a) First Quantitative measure at day 0
  (b) Second Quantitative measure after 6 months
2. The strains announced are present in the product

Companies seeking to obtain the label are committed to providing the information and elements necessary for the successful completion of its mission within the specified time.

The Scientific Committee includes a panel of experts which, after receiving the results from the mandated Laboratory, decides whether to grant or refuse the ESLP label for the submitted product. Besides, the product quality of the probiotics granted with the ESLP Quality Label is rechecked on a regular basis by the accredited lab on specific demand from the ESLP.

## 4. Relevance of the Accreditation in Probiotic Testing

In Europe, an international accreditation system is in place through the activity of several national bodies that together report to the European Accreditation body EA. These bodies, such as ACCREDIA in Italy and BELAC in Belgium, assess, through yearly auditing visits, that independent laboratories can perform the accredited tests as required by the relevant standards and methods. In other terms, accreditation bodies certify that testing laboratories “operate in accordance with the provisions of the norm ISO 17025 means to possess the technical expertise to carry out the analysis, a system of quality management, essential to ensure the accuracy of the analytical data, to guarantee the traceability of measurements and the compliance of own expertise to the international regulatory requirements as well as to the mandatory legislative requirements” (ISO 17025:2017) [23].

The experience of independently accredited laboratories is paradigmatic in the relevance of the assessment of the quality of probiotic preparations. In fact, in a recent publication issued by the International Scientific Association for Probiotics and Prebiotics [4,6], the assignment of product testing to specialized audited laboratories is clearly stated by several international organizations involved in third-party certification. Moreover, analytical methods should be robust in terms of reproducibility and repeatability and, especially in the case of multi-strain preparations, validated under the provisions of ISO 16140 [24] with the uncertainty calculated as indicated in ISO 19036 [25].

## 5. Supranational Collaborations in Probiotics Testing

Laboratories offering a quality service for the microbiological enumeration of bacteria in probiotic products must implement a quality assurance system. An effective system should, in addition to daily quality control of procedures, consumables, equipment, and personnel, include the use of validated test methods, and participation in a proficiency testing scheme and/or inter-laboratory comparative trial.

Previous experiences demonstrated that spontaneous supranational collaborations between independent third-party laboratories are possible and should be promoted, such as the case of the trial between 15 laboratories that led to an international standard on the application of flow cytometry to the quantification of lactic acid bacteria (ISO 19344:2015 IDF 232:2015) [26]. The flow cytometry technology was applied to the quantification and viability assessment of microorganisms in multi-strains probiotic products [27] and in finished product formulation containing a single probiotic strain [28].

In the attempt to explore the opportunity to extend ESLP experience to other EU countries and to identify the key issues of the supranational evolution of the initiative, the methodological biases were identified as one of the most urgent problems. Microbes’ enumeration by CFU is still the gold standard approach to quantify viable cells in probiotic products, despite many challenges associated with plate count methods. Reputed independent accredited laboratories Quality Partner (Herstal, Belgium) and AAT-Advanced Analytical Technologies (Fiorenzuola d’Arda (Piacenza), Italy) were taking part in a comparative trial, involving the species-specific quantification of viable probiotics in finished commercial multi-strain probiotic preparations. The aim of the inter-laboratory comparative trial was to determine and compare the performance of the two laboratories in probiotics enumeration by plate count approach. A total of four commercial probiotic food supplements (two collected from the Italian market and two from the Belgian market) were analyzed by Quality Partner (QP) and AAT laboratories (Table 1). Please note that the lactobacilli taxonomy has changed [29], but it is still in a transition period; so, we have chosen to still use in Tables the former nomenclature. All commercial, finished products contained a combination of two probiotic strains belonging to the following species: *Lactobacillus acidophilus*, *Bifidobacterium animalis* subsp. *lactis* and *Lactobacillus casei*. The most important feature of probiotic products is the viability and accurate bacteria contents mentioned on the label. The quantification of bacteria was based on the culture results from the selective media for each tested bacterial species/strain. ISO methods and a method reported on an Italian technical document were applied. In Table 2 are reported the selective growth media and culturing conditions used by both laboratories for probiotic quantification. The probiotic food supplements were analyzed by three technicians per each laboratory (two plates per dilution were inoculated), and the final viable counts were calculated using the formula reported in Annex D of ISO 7218:2017/Amd.1:2013 [30]. The following statistical parameters were calculated from the results of each sample: mean, standard deviation, coefficient of variation, the percent relative uncertainty, and accuracy. The assessment of laboratories’ performances was evaluated using the *z*-score parameter. The *z*-score values were calculated using the formula reported in ISO 13528:2015 [31] and the related conventional interpretation criteria.

**Figure 1:**
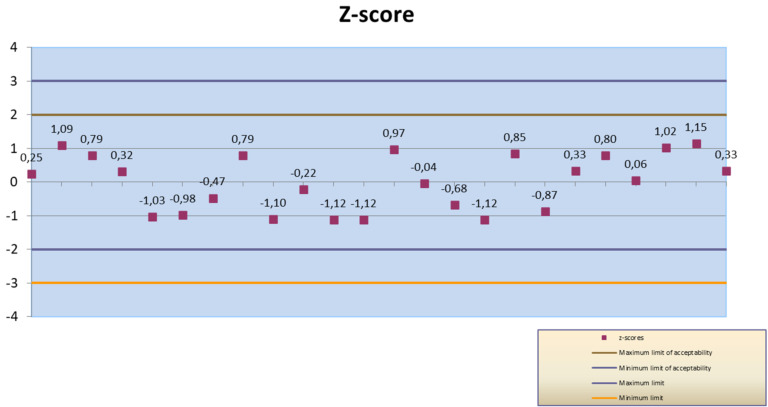
*Z*-score values obtained by operators of Lab1.

**Figure 2:**
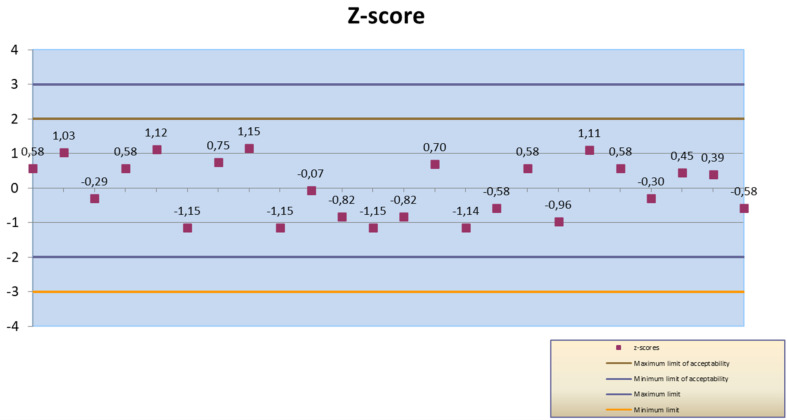
*Z*-score values obtained by operators of Lab2.

The culturing technique allowed for the determination of the bacterial viability and for a count of the number of CFU per each bacterial ingredient in the commercial products. The CFU counts of each probiotic strain were reported in Table 3. The *z*-score value obtained for all participant and for each laboratory were plotted in Figure 1 and Figure 2 and the limits added (|*z*| ≤ 2 Satisfactory, 2 < |*z*| < 3 Questionable, |*z*| ≥ 3 Unsatisfactory). The *z*-score of all participants resulted to be satisfactory (*z*-score < ± 2).

## 6. Sharing the Experience and Developing Common Approaches

This manuscript is aimed to share with readers the experience of internationalization experienced by two relevant EU laboratories in defining and applying common technical approaches in evaluating the quality of probiotic preparations. The relevance of this topic is demonstrated by the number of publications recently issued and reported below and by the active international debate on probiotics and their regulatory framework. Moreover, promoting this type of collaboration will reassure and convince consumers of the management of this issue related to the quality and labeling of probiotics.

The ESLP is today implementing the ESLP Quality Label internationalization in Europe and will celebrate in 2021 the 10th year anniversary of the ESLP Quality Label. This initiative supports the recent recommendations from the International Scientific Associations, all parties involved agreeing on the need for a “Quality Label” for qualified products.

1. The ISAPP [35] recent call in Frontiers in Microbiology for Improving end-user trust in the quality of commercial probiotic products, with reference to the ESLP Quality Label experience in Europe [6].
2. the ESPGHAN [7] working group for Probiotics and Prebiotics recommending the minimum criteria for probiotics:
  a being sufficiently characterized,
  b safe,
  c supported by at least one positive trial according to generally accepted scientific standards—the beneficial effects of probiotics being strain specific; not all the positive results of one strain or association can be generalized to other strains or associations
  d alive in adequate numbers in the product throughout shelf-life and when consumed [7]
3. The ISAPP and IPA Criteria to Qualify Microorganisms as “Probiotic” in Foods and Dietary Supplements with respect to commercial communications defending the same principles [6].

## 7. Future Perspectives of Quality Assessment of Probiotics

In the probiotic industry, the assessment of viability (live microorganisms) is mainly referred to the “enumeration by plate count technique” of the selected microorganisms. This conventional approach is still the gold-standard to quantify viable bacteria in probiotic preparations, and the results generated are expressed as the Colony Forming Unit (CFU). However, the cultivability is only a subset of the possible “viable” status of a bacterial cell, and plating assays might underestimate the microbial potency when the cells are in “viable but not cultivable state” (VBNC). Moreover, the development of specific growth media for specific microorganisms are often time consuming, and as the probiotics market evolve, this development will become more and more complicated. Molecular techniques (metagenomic, qPCR, flow cytometry) are under use in the field of probiotics quality and quantity assessment. However, these techniques as alternative or complementary to conventional microbiology show a real and deeper efficiency. They must be integrated as routine analysis for some specific products with a complex formulation, whose presence on the EU market is constantly increasing.

Enumeration of bacteria is not the only aspect connected to the quality assessment of probiotic strains. Upstream of this technical aspect, it is necessary to define the criteria to qualify microorganisms as “probiotic strain”. Recently, Binda and collaborators (2020) suggested the minimum criteria needed for the proper use of the term probiotic, upgrading the previous assessments by Hill et al. (2014) [4,16]. Specifically, the authors proposed a decision tree of activities to determine if a candidate probiotic strain fulfills the definition criteria. The characterization of probiotic strains should support their probiotic physiological activity while clinical outcomes are necessary for a claim of probiotic functionality.

Related to the future perspectives of quality assessment, it also concerns not only the next generation probiotics but also the new probiotic-related concepts such as postbiotics and paraprobiotics emerging and getting more and more interest from the industry and the consumers.

Concerning the new generation of probiotics, we are not dealing with lactic acid bacteria, but we refer to a new generation of bacteria, generally not included in the QPS list, that appear very promising for their impact on human health but that will need a novel food approval in the EU. These bacterial genera and species present many challenges: organisms not easy to culture, high sensitivity to oxygen, high range of taxonomic diversity, limited information available in the literature regarding their preservation. Taken together, all these features make up a picture of considerable complexity and many challenges to be able to characterize the quality of the finished products. Accredited laboratories must improve their instrumentation to meet the stringent requirements of these new strictly anaerobic genera and their procedures to assure reliable culturing, quantification, and identification.

The new probiotic-related concepts such parabiotics/paraprobiotics and postbiotics are indicating that non-viable microorganisms or bacterial-free extracts may provide benefits to the host by offering additional bioactivities to viable probiotics.

Paraprobiotics are non-viable probiotics or inactivated probiotics or ghost probiotics, which, when administered in sufficient amounts, confer benefits to the host.

The tentative term Postbiotics has been the most used one so far and is increasingly found in the scientific literature and on commercial products, yet is inconsistently used and lacks a clear definition. Postbiotics are compounds produced by microbes released from food components or microbial constituents, including non-viable cells that, when administered in adequate amounts, promote health and wellbeing [36].

There is increasing evidence on the health effects of non-viable microorganisms and the metabolites that they can produce by fermentation or by their action on food components.

Different terms have been used in the literature to refer to these bioactive compounds, which do not fall under the known categories of probiotics, prebiotics, or synbiotics.

In 2019, a panel of experts specializing in nutrition, microbial physiology, gastroenterology, pediatrics, food science, and microbiology from the International Scientific Association for Probiotics and Prebiotics (ISAPP) did review the definition and scope of postbiotics. The panel defined a postbiotic as a “preparation of inanimate microorganisms and/or their components that confers a health benefit on the host”. Effective postbiotics must contain inactivated microbial cells or cell components, with or without metabolites, that contribute to observed health benefits [37].

The diversification of the types of products based on probiotics, conventional, or of new generation, live but also inactivated, cell fractions and metabolites, confirms the need for a joint commitment of accredited laboratories at the European level for the identification and validation of reliable systems for the assessment of the quality of these complex and composite products.

## Figures and Tables

**Table 1:** List of probiotic supplements.

Market	Probiotic	Packaging	Label Composition ^a^	Expiration Date ^a^
Belgium	A	Capsule	12.5 billion CFU/cps of *L. acidophilus,* 12.5 billion CFU/cps of *B. animalis* subsp. *lactis*	01/2021
	B	Capsule	5 billion CFU/cps of *L. acidophilus,* 5 billion CFU/cps of *B. animalis* subsp. *lactis*	06/2021
Italy	C	Sachet	1.5 billion CFU/sachet of *L. acidophilus,* 1.5 billion CFU/sachet of *B. animalis* subsp. *lactis*	10/2020
	D	Capsule	2 billion CFU/cps of *L. acidophilus,* 1 billion CFU/cps of *L. casei*	07/2020

^a^ Information labeled on probiotics products, CFU: colony-forming unit, cps=capsule.

**Table 2:** Selective growth media and culturing conditions.

Bacterial Target	Method of Analysis	Medium Culture and Supplement	Diluent	Growth Condition
*L. acidophilus*	ISO 20128:2006 [32]	MRS Clindamycin 0.1 µg/mL and Ciprofloxacin 10 µg/mL	MRD (Maximum Recovery Diluent)	Anaerobic incubation at 37 °C for 72 h
*B. animalis lactis*	ISO 29981:2010 [33]	TOS Propionate Mupirocin 50 µg/mL		
*L. casei*	Rapporti ISTISAN 2008/36 [34]	MRS Vancomycin 10 µg/mL		

**Table 3:** Plate count of probiotic bacteria in commercial products: mean, standard deviation, coefficient of variation, percent relative uncertainty, *z*-score, and accuracy.

Product		Sample A		Sample B		Sample C		Sample D	
Parameter		*Lactobacillus acidophilus*	*Bifidobacterium lactis*	*Lactobacillus acidophilus*	*Bifidobacterium lactis*	*Lactobacillus acidophilus*	*Bifidobacterium lactis*	*Lactobacillus acidophilus*	*Lactobacillus casei*
Value stated by producer (Log10 CFU/g)		**10.67**	**10.67**	**10.52**	**10.52**	**9.10**	**9.10**	**9.78**	**9.48**
**LAB 1**	Operator 1 (log10 CFU/g)	10.56	10.18	10.60	10.15	9.43	8.58	10.15	10.74
	Operator 2 (log10 CFU/g)	9.92	9.65	10.08	9.83	9.88	9.04	10.08	10.08
	Operator 3 (log10 CFU/g)	10.85	9.40	10.48	10.26	9.67	9.56	10.68	10.58
**LAB 2**	Operator 1 (log10 CFU/g)	10.69	10.74	10.18	10.36	9.36	9.73	10.77	10.28
	Operator 2 (log10 CFU/g)	10.65	10.71	10.15	10.32	9.28	9.79	10.73	10.18
	Operator 3 (log10 CFU/g)	10.69	10.68	10.26	10.36	9.30	9.78	10.76	10.18
**Precision**	LAB 1 Mean	10.44	9.74	10.39	10.08	9.66	9.06	10.30	10.47
	LAB 1 SD	0.47	0.40	0.27	0.22	0.22	0.49	0.33	0.34
	LAB 1 Standard Error	0.08	0.07	0.05	0.04	0.04	0.09	0.06	0.06
	K2 uncertainty with 95% confidence	9.08%	8.14%	5.26%	4.43%	4.60%	10.78%	6.41%	6.59%
	*Z*-score 1	0.25	1.09	0.79	0.32	−1.03	−0.98	−0.47	0.79
	*Z*-score 2	−1.10	−0.22	−1.12	−1.12	0.97	−0.04	−0.68	−1.12
	*Z*-score 3	0.85	−0.87	0.33	0.80	0.06	1.02	1.15	0.33
	Variation coefficient	4.538%	4.072%	2.629%	2.214%	2.299%	5.392%	3.203%	3.295%
	LAB 2 Mean	10.68	10.71	10.19	10.35	9.31	9.77	10.76	10.21
	LAB 2 SD	0.02	0.03	0.06	0.02	0.04	0.03	0.02	0.06
	LAB 2 Standard Error	0.00	0.01	0.01	0.00	0.01	0.01	0.00	0.01
	K2 uncertainty with 95% confidence	0.40%	0.55%	1.11%	0.44%	0.92%	0.59%	0.38%	1.16%
	*Z*-score 1	0.58	1.03	−0.29	0.58	1.12	−1.15	0.75	1.15
	*Z*-score 2	−1.15	−0.07	−0.82	−1.15	−0.82	0.70	−1.14	−0.58
	*Z*-score 3	0.58	−0.96	1.11	0.58	−0.30	0.45	0.39	−0.58
	Variation coefficient	0.2000%	0.2766%	0.5533%	0.2204%	0.4611%	0.2941%	0.1897%	0.5805%
**Accuracy**	Accuracy LAB 1	−0.23	−0.92	−0.14	−0.45	0.56	−0.04	0.52	0.98
	Accuracy LAB 2	0.01	0.04	−0.33	−0.17	0.22	0.67	0.97	0.73

## Data Availability

Not applicable.
